# One test for all: whole exome sequencing significantly improves the diagnostic yield in growth retarded patients referred for molecular testing for Silver–Russell syndrome

**DOI:** 10.1186/s13023-021-01683-x

**Published:** 2021-01-22

**Authors:** Robert Meyer, Matthias Begemann, Christian Thomas Hübner, Daniela Dey, Alma Kuechler, Magdeldin Elgizouli, Ulrike Schara, Laima Ambrozaityte, Birute Burnyte, Carmen Schröder, Asmaa Kenawy, Peter Kroisel, Stephanie Demuth, Gyorgy Fekete, Thomas Opladen, Miriam Elbracht, Thomas Eggermann

**Affiliations:** 1 Institute of Human Genetics, Medical Faculty, RWTH Aachen University, Pauwelsstr. 30, 52074 Aachen, Germany; 2 Institute of Human Genetics, University Hospital Essen, University Duisburg-Essen, Essen, Germany; 3 Department of Neuropediatrics, University Children’s Hospital, University Duisburg-Essen, Essen, Germany; 4 Department of Human and Medical Genetics, Institute of Biomedical Sciences, Faculty of Medicine, Vilnius University, Vilnius, Lithuania; 5 Zentrum Für Kinder- Und Jugendmedizin, Abt. Allgemeine Pädiatrie, Universitätsmedizin Greifswald, Greifswald, Germany; 6 Department of Human Genetics, Medical Research Institute, Alexandria University, Alexandria, Egypt; 7 Institute of Human Genetics, Graz, Austria; 8 Praxis Für Humangenetik, Erfurt, Germany; 9 II. Department of Pediatrics, Semmelweis University, Budapest, Hungary; 10 Division for Child Neurology and Metabolic Medicine, University Children’s Hospital Heidelberg, Heidelberg, Germany

**Keywords:** Silver–Russell syndrome, Next generation sequencing, Diagnostic detection rate, Whole exome sequencing, Targeted multigene panel NGS

## Abstract

**Background:**

Silver-Russell syndrome (SRS) is an imprinting disorder which is characterised by severe primordial growth retardation, relative macrocephaly and a typical facial gestalt. The clinical heterogeneity of SRS is reflected by a broad spectrum of molecular changes with hypomethylation in 11p15 and maternal uniparental disomy of chromosome 7 (upd(7)mat) as the most frequent findings. Monogenetic causes are rare, but a clinical overlap with numerous other disorders has been reported. However, a comprehensive overview on the contribution of mutations in differential diagnostic genes to phenotypes reminiscent to SRS is missing due to the lack of appropriate tests. With the implementation of next generation sequencing (NGS) tools this limitation can now be circumvented.

**Main body:**

We analysed 75 patients referred for molecular testing for SRS by a NGS-based multigene panel, whole exome sequencing (WES), and trio-based WES. In 21/75 patients a disease-causing variant could be identified among them variants in known SRS genes (*IGF2, PLAG1, HMGA2*). Several patients carried variants in genes which have not yet been considered as differential diagnoses of SRS.

**Conclusions:**

WES approaches significantly increase the diagnostic yield in patients referred for SRS testing. Several of the identified monogenetic disorders have a major impact on clinical management and genetic counseling.

## Background

Growth retardation is a common condition and often manifests during pregnancy. Though several non-genetic factors have been identified, a significant number of patients with growth retardation carry genetic alterations. Severe intrauterine and postnatal growth failure is a typical feature of Silver-Russell syndrome (SRS, OMIM#180860), a congenital disorder which is additionally characterised by relative macrocephaly (head circumference at birth ≥ 1.5 SDS above birth weight and/or length SDS), asymmetry, a typical triangular face with a prominent forehead, and feeding difficulties. However, due to the low specificity and heterogeneity of features the clinical diagnosis of SRS is difficult, and therefore the Netchine–Harbison clinical scoring system (NH-CSS) has been suggested to standardise the subjective clinical diagnosis [1, 2] (see Additional file 1: Table 1). Additionally, the clinical diagnosis is complicated by the lack of specificity of several symptoms and the phenotypic overlap with other (growth retardation) disorders.

**Table 1:** Overview on the molecular and major clinical findings in the 15 newly identified patients carrying pathogenic variants. For further details see supplementary patients descriptions (ACMG American College of Medical Genetics and Genomics [9]; VUS Variant of Unknown Significance; SDS standard deviation score; ND not determined; NA not available; gw gestational week; DD developmental delay; ad autosomal dominant, ar autosomal recessive; hom homozygous, het heterozygous, hem hemizygous, com het compound heterozygous, imp imprinted; pat paternal, mat maternal; *published by Hübner et al. [17], ** published by Meyer et al. [25, 26]; *** according to the ACMG guidelines, these variants should be classified as VUS. However, in this study they were classified as (likely) pathogenic, see Additional file 3: Table 3)

Gene	Patient	OMIM*	Sex	gw	Weight at birth: g (SDS)	Length at birth: cm (SDS)	OFC at birth: cm (SDS)	Age at examination	Weight at examination: kg (SDS)	Height at examination: cm (SDS)	OFC at examination: cm (SDS)	Protruding forehead	Asymmetry
*PLAG1*	**1**	603026	F	36	1370 (−3.12)	39 (−3.12)	30.5 (−2.2)	1 7/12 years	7.4 (−3.0)	71 (−3.6)	45 (−2.07)	Yes	No
*IGF2*	**2a**	147470	F	37	1800 (−2.7)	45 (−1.87)	32 (−1.36)	1 10/12 years	7.4 (−3.62)	74 (−3.5)	44 (−3.75)	Yes	Yes
	**2b**		F	37	1590 (−3.18)	43 (−2.71)	31 (−2.07)	NA	NA	NA	NA	Yes	Yes
*HMGA2*	3*	600698	M	39	2360 (−2.6)	47 (−2.13)	31 (−3.31)	3 8/12 years	10 (−3.93)	88 (−3.24)	NA	Yes	No
*HMGA2*	4a*	600698	F	39	1400 (−4.65)	36 (−6.86)	NA	3 years	6200 (−7.09)	70 (−6.73)	42.5 (−7.42)	Yes	Uncertain
	4b*		M	38	1050 (−5.18)	NA	NA	1 1/12 years	4100 (−6.76)	57 (−6.19)	40 (−6.06)	Yes	Uncertain
*IGF1R*	**5**	147370	F	31	930 (−2.03)	37 (−1.45)	NA	1 11/12 years	6.12 (−5.0)	74 (−3.5)	39 (−9.79)	Yes	No
*ORC1*	**6**	601902	F	40	1435 (−4.88)	43 (−4.02)	30 (−3.84)	31 years	21 (−10.31)	137 (−4.7)	47.5 (−7.15)	No	No
*OBSL1*	**7**	610991	M	NA	NA	NA	NA	2 5/12 years	8.3 (−4.02)	71.5 (−5.69)	50 (−0.16)	Yes	Yes
*MBTPS1*	8**	603355	M	40	2400 (−2.78)	47 (−2.39)	35 (−0.46)	5 4/12 years	120 (-4.51)	98.5 (-3.19)	49 (−2.21)	No	No
*FANCA*	**9**	607139	F	35	1099 (−2.98)	38 (−3.0)	27.5 (−2.88)	3 3/12 years	10.28 (−3.23)	85 (−3.23)	44 (−5.35)	Yes	No
*NF1*	**10**	613113	M	25.5	620 (−1.33)	32 (−0.7)	22.5 (−1.11)	6 2/12 years	12.65 (−5.08)	105 (−2.86)	47.5 (−3.65)	Yes	No
*FGD1*	**11**	300546	M	40	2700 (−2.13)	48 (−1.99)	33 (−2.03)	13 3/12 years	38 (−1.26)	148.8 (−1.99)	52 (−2.11)	Yes	No
*CNOT3*	12**	604910	F	40	3200 (−0.65)	48 (−1.68)	35 (0.18)	NA	NA	NA	NA	No	No
*KMT2C*	**13**	606833	M	41	3.35 (−0.85)	53 (−0.04)	35 (−0.69)	13 3/12 years	25.6 (−3.5)	138 (−2.7)	51 (−2.82)	No	No
*PTEN*	**14**	601728	M	38	3700 (0.89)	56 (2.13)	36 (0.79)	10 5/12 years	39 (0.53)	141 (−0.35)	61 (5.29)	No	No
*PTPN11*	**15**	176876	M	NA	NA	NA	NA	15 11/12 years	44.5 (−2.37)	161.1 (−2.03)	NA	No	Yes

Gene	Patient	Feeding difficulties	NH CSS	Coding DNA level	Genomic DNA level (GRCh38)	Protein level	Variant already reported	Mendelian trait	Inheritance	Family members affected	ACMG classification	Associated syndrome	OMIM#
*PLAG1*	**1**	Yes	4/6	NM_002655.2: c.599dup	Chr8: g.56167147dup	p.(Arg201Profs*52)	No	ad, het	de-novo	no	pathogenic: PVS1, PM2, PM6	SRS	180860
*IGF2*	**2a**	Yes	5/6	NM_001127598: c.381 T > G	Chr11: g.2133610A > C	p.(Cys127Trp)	No	ad, het, imp	pat	sister	VUS***: PM2, PP3, PP1	SRS	180860
	**2b**	Yes	4/4										
*HMGA2*	3*	Yes	4/6	NM_003483.4: c.111 + 1G > T	Chr12: g.65825382G > T	p.?	See Additional file 3: Table 3	ad, het	de-novo	no	pathogenic: PVS1, PM6, PM2	SRS	180860
*HMGA2*	4a*	Yes	4/4	NM_003483.4: c.239C > T	Chr12: g.65838559C > T	p.(Pro80Leu)	See Additional file 3: Table 3	ar, hom	mat/pat	brother	VUS***: PM2, PP3	SRS	180860
	4b*	Yes	4/4										
*IGF1R*	**5**	No	3/6	NM_000875: c.3530G > A	Chr15: g.98942995G > A	p.(Arg1177His)	See suppl. Information	ar/ad, het	Pat	NA	likely pathogenic: PS2, PM2, PP3	resistance to IGF1	270450
*ORC1*	**6**	Yes	3/6	NM_004153.3: c1996C > T c.692del	Chr1: g.52383437G > A g.52861747del	p.(Arg666Trp) p.(Pro231Glnfs*12)	rs201253919 rs1362231446	ar, com het	mat/pat	no	c.1996C > T: likely pathogenic: PM2, PM3, PP3, PP5 c.692del: likely pathogenic: PVS1, PM2	Meier-Gorlin syndrome 1	224690
*OBSL1*	**7**	Yes	4/6	NM_015311.2: c.1382G > A arr[hg19]2q35 (220300895_220596562) × 1	Chr2: g.219567870C > T	p.(Trp461*) whole gene deletion	No	ar, com he	NA	NA	pathogenic: PVS1, PM2, PM3	3 M syndrome 2	612921
*MBTPS1*	8**	Yes	4/6	NM_003791.3: c.1094A > G	Chr16: g.84087398 T > C	p.(Asp365Gly)	rs1226321681	ar, hom	mat/pat	No	likely pathogenic: PS3, PM2, PP3, PP5	Spondyloepiphyseal dysplasia	618392
*FANCA*	**9**	Yes	4/6	NM_000135.2: c.2851C > T c.2222 + 1G > T	Chr16: g.89761950G > A g.89770563:C > A	p.(Arg951Trp) p.?	rs755546887, rs775488912	ar, com het	mat/NA	no	c.2851C > T: likely pathogenic: PM2, PM3, PP5, PP3 c.2222 + 1G > T: likely pathogenic: PVS1, PM2	Fanconi anemia, complementation group A	227650
*NF1*	**10**	Yes	3/5	NM_001042492:c.5488C > T	Chr17: g.31327718C > T	p.(Arg1830Cys)	see suppl. Inform	ad, het	mat	no	likely pathogenic: PS3, PM2, PP3, PP5	NF1	162200
*FGD1*	**11**	NR	2/5	NM_00463.2: c.2761C > T	ChrX: g.54446234G > A	p.(Arg921*)	rs869312743	XLR, hemi	de-novo	NA	likely pathogenic: PS2, PM2	Aarskog-Skott syndrome	305400
*CNOT3*	12**	Yes	2/5	NM_014516.3: c.658G > T	Chr19: g.54145772G > T	p.(Glu220*)	No	ad, het	de-novo	two children	pathogenic: PVS1, PS2, PM2, PP3	Developmental disorder, speech delay, autism, dysmorphic facies	618672
*KMT2C*	**13**	No	1/6	NM_170606.2: c.11023C > T	Chr7: g.151859639G > A	p.(Gln3675*)	No	ad, het	de-novo	no	pathogenic: PVS1, PM2, PM6	Kleefstra syndrome 2	617768
*PTEN*	**14**	No	1/5	NM_000314.7: c.518G > A	Chr10: g.87952143G > A	p.(Arg173His)	rs121913294	ND	ND	no	likely pathogenic: PM1, PM2, PP3, PP5	Macrocephaly/Autism	605309
*PTPN11*	**15**	No	2/2	NM_002834.4: c.1508G > A	Chr12: g.112489084G > A	p.(Gly503Glu)	rs397507546	ad	ND	ND	likely pathogenic: PM1, PM2, PP3, PP5	Noonan?	163950

The clinical heterogeneity of SRS is reflected by a broad spectrum of molecular disturbances. With nearly 40%, the major finding in patients with the typical SRS phenotype is loss of methylation (LOM) of the imprinting centre region 1 (IC1, *H19/IGF2*:IG-DMR) in 11p15.5 (for review: [2]). Up to 10% of SRS patients exhibit a maternal uniparental disomy of chromosome 7 (upd(7)mat), and a relevant number of patients carry alterations of chromosome 14q32 which are typically associated with Temple syndrome (TS14, OMIM#616222). In fact, TS14 patients show a clinical overlap with SRS, but both carriers of upd(7)mat and 14q32 alterations exhibit a less typical SRS phenotype compared to patients with an IC1-LOM [1, 3]. Nevertheless, even in the IC1-LOM group the clinical picture is variable. Additional molecular alterations in SRS comprise less common genetic variants, i.e. UPDs of chromosome 20, diverse submicroscopic deletions and duplications (copy number variants, CNV) [4], and pathogenic variants in single genes. These molecular changes are either associated with typical SRS phenotypes (e.g. microdeletions in 12q14, pathogenic variants in *IGF2, CDKN1C, PLAG1, HMGA2*) [4–6], or with differential diagnoses of SRS (see [2]).

Both clinical and molecular heterogeneity make the decision on the diagnostic procedure in SRS challenging. To address this issue, a stepwise testing procedure has been consented, starting with IC1 LOM analysis as the first step, followed by testing for upd(7)mat, 14q32 and CNVs [2]. However, the subsequent testing strategies have not yet been defined, and the knowledge on further SRS (spectrum) causing genes or associated genomic variants are mainly based on single reports as systematic screening studies aiming on the contribution of monogenic variants to the molecular SRS spectrum are still missing. With the implementation of next generation sequencing (NGS)-based assays in genetic testing, comprehensive approaches to address a broad spectrum of monogenetic diseases have become available. Our group has recently demonstrated the suitability of targeted multigene NGS panels to increase the diagnostic yield in patients with SRS features [7], and first data from whole exome sequencing were promising as well [8].

We now report on the results of a systematic WES approach in a cohort of growth-retarded patients referred for SRS diagnostics, and compare the results with targeted multigene NGS panel data in the same cohort.

### Study cohort

The total cohort consisted of 75 patients with SRS but negatively tested for the typical molecular SRS findings (IC1 LOM, upd(7)mat, 14q32 alterations). WES data were available from 60 patients, the remaining 15 datasets were taken from a targeted NGS approach published recently [7]. In addition to 14 patients not previously analysed by NGS approaches, the cohort included patients already analysed with a targeted NGS approach (n = 47) [7] and from a recent pilot WES study (n = 45) [8], which also partially overlaps (n = 31).

In 22 of these patients, the parents were also analysed by WES (trio-based WES).

To determine the detection rate by the three strategies—targeted NGS, index-based WES and trio-based WES—datasets from all 75 patients were included.

Clinical scoring was leaned on the NH-CSS [1, 2]. The study was approved by the ethical committee of the Medical Faculty of the RWTH Aachen (EK303-18).

## Materials and methods

The first step molecular diagnostic testing in all patients comprised analyses of IC1 LOM and upd(7)mat analysis by methylation-sensitive multiplex ligation-dependent probe amplification assays, which also covers 14q32 alterations (MS-MLPA)(ME030, ME032; MRC Holland, Amsterdam/NL). This initial screening was conducted by the authors or by external laboratories. After exclusion of these (epi)mutations, all samples were screened for upd(20)mat, upd(16)mat and submicroscopic copy number variations (> 50 kb) by microsatellite typing, MS-MLPA (ME034), MS single nucleotide primer extension and SNP array analysis (deletions/duplications > 50 kb, CytoScan® HD Array (Affymetrix, Santa Clara/CA, USA)). Nearly all patients were negatively tested with one exception with an *OBSL1* microdeletion (patient 7).

For whole exome sequencing of the index patients and their parents DNA samples isolated from peripheral blood were enriched using the Nextera Rapid Capture Exome (v.1.2) (Illumina, San Diego, CA, USA). The enriched libraries were sequenced on a NextSeq500 Sequencer with 2 × 75 cycles on a high-output flow cell. Fastq file generation and adapter trimming was performed using bcl2fastq2 (Illumina, San Diego, CA, USA). The automated SeqMule pipeline (v1.2.6) was used for fastq quality assessment (FastQC, v.0.11.2), alignment (BWA-MEM, v.0.7.8-r455), duplicate removal (SAMtools, rmdup, v.0.1.19–44,428 cd), Indel Realignment (GATKLite realign; v.2.3–9)) and variant calling. Three different variant callers were used (GATKLite, (UnifiedGenotyper version: 2.3–9), SAMtools (mpileup; v.0.1.19–44428 cd), FreeBayes (v.0.9.14–14-gb00b735)) for variant detection, and variants shared by at least two out of three variant callers were considered for further analysis. Annotation and bioinformatic prioritization of variants was performed using KGGSeq (v1.0, 20/Jun./2018). Variants with a minor allele frequency (MAF) higher than 0.75% in public databases (i.e. gnomAD, EXAC, 1000 GP, ESP) and synonymous variants were excluded. Sequencing variants were classified according to the ACMG criteria [9]. Variants identified as pathogenic or likely pathogenic were confirmed by Sanger sequencing on an ABI3500 platform (Applied Biosystems, Foster City, CA, USA) and segregation analysis in the family was performed if possible.

WES data were analyzed for variants in the genes included in the panel approach (for the list of genes see Additional file 2: Table 2 [7]). The achieved WES data from our cohort were filtered for pathogenic variants in all genes listed in OMIM. In 22 patients, parental DNA samples could be used for trio WES analysis. The comparison of detection rates of the three strategies was then restricted to the subcohort from [7] as these patients were analyzed by all three approaches.

## Results

In a cohort of 75 patients referred for SRS testing, NGS data were analysed to identify the disease-causing genetic alterations. WES data were available for 59 patients: For 14 patients these datasets were newly generated, for the remaining 45 cases data from a recent study were compiled [8]. In 16 patients, only panel-based NGS data were considered. In 22 out of the 59 patients analysed by WES, parental samples were available for trio-based analysis.

In the total cohort, pathogenic variants explaining the growth retardation phenotypes could be detected in 21 cases (28.0%), six of them had been published recently [7, 8]. The molecular and clinical data of the newly identified 15 patients are listed in Table 1 and a short description is given in the Additional file 3: Table 3.

**Fig. 1:**
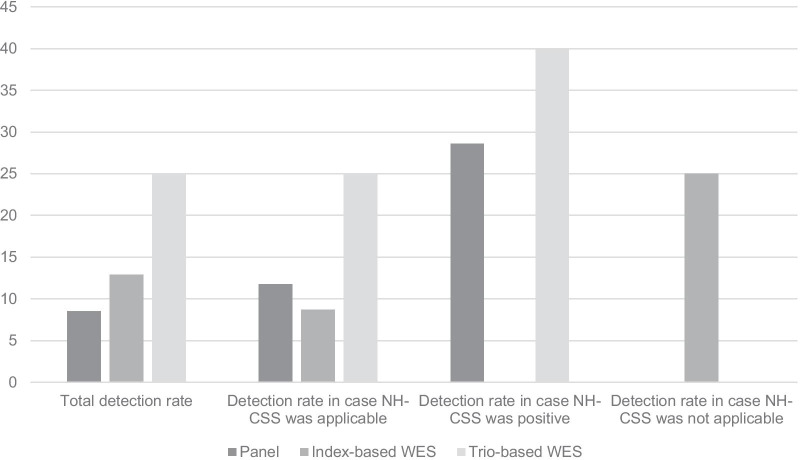
Detection rates of different NGS approaches in the cohort of patients referred for SRS testing, and discrimination of the diagnostic yields between patients for whom the clinical score was applicable, and NH-CSS positive cases (NH-CSS ≥ 4 items) and patients without sufficient clinical data (for number see Additional file 4). Comparison of the detection rates between the multigene panel approach [7] with those obtained by WES approaches revealed a significant increase in patients with a positive NH-CSS score as we well as in patients for whom clinical scoring was not possible

For comparison of the diagnostic yield of the different approaches, detection rates of the WES (index only) and WES trio strategies were restricted to those 47 patients originally analysed with a targeted multigene panel by Meyer et al. [7] (Fig. 1). In 31 negatively tested samples from this subcohort, WES was performed, and allowed the identification of four genetic variants (4 out of 31 patients: 12.9%), affecting the genes *ORC1, FDG1, KMTC2,* and *PTPN11*. Trio analysis in 16 families out of this group revealed four additional pathogenic variants (4 out of 16 families: 25.0%) in the *FANCA, MBTPS1, CNOT3*, and *NF1* genes. In total, a cumulative detection rate of more than 40% could be achieved by this step-wise procedure.

The correlation of the clinical data with the genetic findings confirmed that patients carrying mutations in genes associated with SRS generally showed a higher NH-CSS score (*PLAG1* in patient 1, *IGF2* in patient 5a and her sister 5b, *HMGA2* in patient 6). However, despite a scoring of at least 4 out of 6 criteria, none of them exhibited both key features, relative macrocephaly at birth and protruding forehead. Whereas protruding forehead was present in the majority of patients, relative macrocephaly at birth was observed only in the patient with a homozygous *MBTPS1* variant (patient 8). In general, intrauterine and postnatal growth retardation associated with a triangular face were the major reasons for referral to diagnostic testing and led to the clinical diagnosis of SRS, though several patients did not fulfil the NH-CSS criteria.

Though clinical scoring was not applicable in all cases, the correlation between clinical scoring and the testing strategies showed that pathogenic variants in several patients with a positive NH-CSS score could be identified only by (Trio) WES but not by the panel-based approach (Fig. 1).

## Discussion

The identification of molecular causes of growth retardation is hampered by the large number of genetic factors contributing to growth. Even in SRS as a recognizable phenotype the spectrum of pathogenic variants is broad, and only in up to 60% of patients with the typical SRS features the disease-specific molecular alterations (IC1-LOM, upd(7)mat, 14q32 alterations) can be detected [10]. Thus, a significant number of patients remain without a molecular confirmation of their diagnosis, and only some of them might be explained by mosaicism of the IC1-LOM in 11p15.5 which escapes detection in case of an extremely discrepant distribution in the body [11]. In single families pathogenic variants in genes localized in 11p15.5 (*IGF2, CDKN1C*) or members of the HMGA2-PLAG1-IGF2 pathway have been identified [12–14].

The clinical diagnosis of SRS is additionally hampered by the lack of specificity of its major symptoms, and the overlap with other congenital disorders. As array based studies show, several patients with SRS features carry pathogenic submicroscopic copy number variations associated with differential diagnoses of SRS (CNVs) [4]. Accordingly, small variants in differential diagnostic genes have also been described in SRS patients [2, 7]. To encompass this broad range of putative disease-causing genes, NGS based strategies are a valuable tool as they have the potential to cover all genes and genomic variants which might contribute to a specific phenotype. First studies have already confirmed the power of targeted and whole exome NGS approaches to increase the diagnostic yield in growth retarded patients [7, 8, 15, 16].

In our cohort of patients referred for SRS testing, we now substantiate the need of NGS-based assays in the molecular diagnostic workup in this heterogeneous cohort, and in agreement with findings from other heterogeneous disorders we show that the application of WES significantly improves the detection rate (21.41% versus 8.5% in the cohort analysed by Meyer et al. [7]) for comparable hands-on time and costs. As our data show, a further increase can be achieved by trio analysis, both in patients fulfilling the clinical SRS criteria according to the NH-CSS score as well as in clinically unselected patients referred for SRS testing (Fig. 1).

Our molecular findings reflect the molecular heterogeneity in patients referred for SRS testing as we could identify pathogenic variants in (a) already known SRS genes, in (b) genes associated with differential diagnoses of SRS, in (c) genes causing phenotypes overlapping with SRS.

### SRS genes (Table 1a)

Up to now, pathogenic variants which might cause the characteristic SRS phenotype have been identified in four genes. The imprinted genes *CDKN1C* and *IGF2* are localized in 11p15.5, and *HMGA2* and *PLAG1* interact with IGF2 [14]. In our cohort, we identified pathogenic variants in three of them. In two sibs affected by SRS (patients 2a/b), an *IGF2* variant was inherited from the healthy father which corresponds to the paternal expression of the gene. In contrast to *IGF2*, the mode of inheritance of variants in *HMGA2* and *PLAG1* is not influenced by the sex of the parent contributing the affected allele as these genes are not imprinted. To the best of our knowledge, the frameshift variant in patient 1 is the third *PLAG1* variant reported in the literature [14], and its de-novo occurrence supports the autosomal-dominant mode of inheritance. The *HMGA2* variants from our cohort (patients 3, 4a/b) have been reported recently [17]. Clinically, the patients show growth retardation and SRS features, and the NH-CSS scoring ranges between 4 and 5 parameters. Protruding forehead as one of the two key features of SRS was present in all of them, but relative macrocephaly as the second main sign was absent. For *HMGA2*, the clinical findings correspond to cases recently overviewed by Leszenski et al. [18].

### Genes associated with already identified differential diagnoses of SRS (Table 1b)

As it could be expected from previous reports [2], several differential diagnoses have now been identified in our cohort, including a disturbance of *IGF1R* (patient 5), Meier-Gorlin syndrome 1 (MGS1; *ORC1*, patient 6), and 3 M syndrome 2 (*OBSL1*, patient 7). In all these patients, severe postnatal (and intrauterine) growth retardation was reported, and this feature was the reason for inclusion in the study. However, microcephaly is the clinical feature which allows the discrimination between SRS and the majority of its differential diagnosis, in our cohort *IGF1R* disturbances and MGS1 [19, 20]. Nevertheless, it should be noted that the patient with the *OBSL1* variant exhibited relative macrocephaly at least at the age of 2 years 5 months, and our case confirms that 3-M syndrome is a relevant differential diagnosis of SRS [21].

### Genes causing phenotypes overlapping with SRS (Table 1c)

The results from exome wide screening for pathogenic variants in our cohort confirms that there is a broad clinical overlap between SRS and other congenital disorders. Therefore, the restriction to a selection of genes with higher degree of clinical concordance should be discussed, as the application of targeted panel will miss the detection of relevant mutations, in particular in tumour predisposition genes.

The only patient with relative macrocephaly at birth and positive NH-CSS scoring is homozygous for a *MBTPS1* variant (patient 8). Interestingly, the same variant has already been reported in another growth retarded patient for whom the diagnosis of SRS was also discussed [22]. Thus, we suggest to add *MBTPS1*-associated disorders to the list of differential diagnosis of SRS.

In two patients (patients 9 and 10), we identified pathogenic variants in tumour predisposition syndromes. These growth retarded patients were reported to show the typical facial gestalt of SRS, and were born small for gestational age. *FANCA*-associated Fanconi anemia has not yet been reported as differential diagnosis of SRS, but the clinical overlap is obvious. In patient 9 from this report, Fanconi anemia was diagnosed at the age of 11 years 9 months. In contrast, patient 10 with a pathogenic *NF1* variant only showed slight symptoms of neurofibromatosis type 1 but symptoms consistent with SRS. The variant of our patient has already been reported in an *NF1* patient in combination with short stature [23].

In five further patients (patients 11–15), pathogenic variants compatible with their clinical features could be identified. None of the associated disorders has already been suggested as differential diagnosis of SRS, and clinical scoring shows that none of these individuals had the typical SRS picture. All were ascertained due to growth retardation and a facial gestalt described as “triangular”.

The results from our study cohort confirm the strengths of NGS-based strategies to decipher the molecular basis of congenital disorders (for review: [24]), and the comparison between them also demonstrates their chances and challenges. Whereas the increase of the detection rate by WES in comparison to the targeted multigene panel approach does not need further explanation, the reason for an additional chance to detect disease-causing variants by trio analysis needs some further comments:

Trio WES includes additional information and allows phasing of the detected variants. Thereby it enables the detection of true compound-heterozygous and de novo variants. Furthermore, it helps to determine if a variant in an imprinted gene affects the expressed or the silenced copy. The estimation of pathogenicity of variants in imprinted genes requires this further information on inheritance and imprinting status. Even a variant with a severe functional impact might be without clinical relevance when the affected copy of the imprinted gene is silenced (i.e. maternal *IGF2* variants).

The detection of a de novo occurrence of a variant can decisively influence the assessment of its pathogenicity. As stated in the ACMG guidelines, de novo occurrence represents a strong criteria for pathogenicity [9]. Trio WES allows the time-saving determination of the pathogenicity of variants of uncertain significance, and the confirmation of compound heterozygosity and of parenthood which is a key criteria for classification [9].

In fact, our study has several limitations. It is based on a cohort of patients referred for SRS diagnostic, but with only limited clinical information (Additional file 3: Table 3). The study population therefore includes a considerable number of patients who do not fulfill clinical criteria of SRS [1]. However, this situation reflects the routine diagnostic workup of a clinically heterogeneous group of patients, for which clinical data are rarely provided. As a consequence, this dataset does not reflect the relative contribution of different monogenic causes to the SRS phenotype, but it allows to broaden the spectrum of disorders with a clinical overlap with SRS. Interestingly, even three out of four patients with typical SRS alterations (i.e. *PLAG1, IGF2, HMGA2*) would not be diagnosed as “clinical SRS” according to diagnostic criteria consented recently [2]. The identification of two patients with tumor predisposition variants (*FANCA, NF1*) again confirms that the strategy of a broad testing is needed in patients diagnosed with SRS. In these situations, the testing result provides the basis for a more precise therapeutic management, which includes the decision on or against growth hormone treatment and tumor monitoring.

The comparison between the different NGS strategies is also difficult as the targeted multigene panel used in this study does not reflect the current knowledge on genes contributing to the SRS phenotype. In fact, the use of panel based NGS assays avoids the detection of unsolicited findings, but it is only of limited value for heterogeneous disorders like SRS. As we could show, more than half of the pathogenic variants would be missed by application of a targeted gene panel because several of the identified genes were not included in the multigene panel assay. Furthermore, a multigene panel reflects the knowledge at the time of the panel design. As the variant in the SRS gene *PLAG1* shows, it was missed in our targeted NGS approach as at the time of the panel design [7] *PLAG1* had not yet been identified as SRS gene. A targeted multigene panel can certainly be upgraded, and further genes can be added to a new panel version or spiked in into an existing enrichment kit, but samples analysed by a previous version cannot be reanalysed.

We therefore suggest WES as a general wet-lab enrichment strategy. To avoid incidental findings and a huge number of variants of unknown significance, the WES data might be filtered by a step-wise virtual multigene panel. If new disease-causing genes are identified, these data can then be reanalysed without generation of new NGS data. In addition, the WES data can be analysed repeatedly to identify pathogenic variants in differential diagnosis genes which are not in the focus and would be missed by multigene panels. In case WES in a single case is negative, a trio analysis might be considered. Additionally, the upcoming implementation of whole genome sequencing (WGS) in molecular genetic testing trials will show whether the detection rates can be further increased.

## Conclusions

Our data confirm that WES is a suitable tool to achieve a significant increase of the diagnostic yield in patients referred for SRS testing. The data further contribute to the heterogeneous molecular spectrum of SRS and clinically overlapping disorders, the identified cases demonstrate the need for precise molecular confirmation as the basis for precision medicine. The foreseeable diagnostic implementation of WGS, long read sequencing (third generation sequencing) in combination with transcriptomics will allow the identification of all molecular alterations in one analysis.

### Databases

ClinVar: https://www.ncbi.nlm.nih.gov/clinvar/.gnomAD: https://gnomad.broadinstitute.org/. dbSNP: https://www.ncbi.nlm.nih.gov/snp/.

## Supplementary information

**Additional file 1**. **Table 1** NH-CSS parameter (from [2]).

**Additional file 2**. **Table 2** List of genes used for targeted NGS analysis. It should be noted that the list is based on that from Meyer et al. [7] and does not include the recently identified SRS gene PLAG1 [14].

**Additional file 3**. **Table 3**: Overview on the availability of clinical data for the patients from the three different NGS strategies.

**Additional file 4**. **File 4**: Patients description.

## Data Availability

Data are available on request.

## Abbreviations

ACMG: American College of Medical Genetics and Genomics
CNV: Copy number variation
IC1: Imprinting Center 1 in 11p15.5
LOM: Loss of Methylation
NGS: Next Generation Sequencing
NH-CSS: Netchine-Harbison Clinical Scoring System
SRS: Silver-Russell syndrome
UPD: Uniparental Disomy
WES: Whole Exome Sequencing
WGS: Whole Genome Sequencing
