# Supplementary material for: One test for all: whole exome sequencing significantly improves the diagnostic yield in growth retarded patients referred for molecular testing for Silver–Russell syndrome
Source: Orphanet J Rare Dis. 2021 Jan 22;16:42. doi: 10.1186/s13023-021-01683-x (PMC7821667; doi:10.1186/s13023-021-01683-x)
Supplement: Supplementary file 1 — Additional file 1. Table 1 NH-CSS parameter (from [2]). [file 13023_2021_1683_MOESM1_ESM.docx]

**Supplementary File 1**

**Clinical criteria of the Netchine-Harbison Scoring system ^1,2^**

*SGA (birth weight and/or birth length:*

≤−2 SDS for gestational age

*Postnatal growth failure:*

Height at 24 ± 1 months ≤ −2 SDS or height ≤ −2 SDS below mid-parental target height

*Relative macrocephaly at birth*

Head circumference at birth ≥1.5 SDS above birth weight and/or length SDS

*Protruding forehead**

Forehead projecting beyond the facial plane on a side view as a toddler (1–3 years)

*Body asymmetry*

LLD of ≥0.5 cm or arm asymmetry or LLD

*Feeding difficulties and/or low BMI BMI*

≤−2 SDS at 24 months or current use of a feeding tube or cyproheptadine for appetite stimulation

Clinical diagnosis is considered if a patient scores at least four of six from these criteria. If all molecular tests are normal and differential diagnoses have been ruled out, patients scoring at least four of six criteria, including both prominent forehead and relative macrocephaly should be diagnosed as clinical Silver–Russell syndrome. *Protruding forehead is equivalent to ‘prominent forehead’. LLD, leg length discrepancy; SDS, SD score; SGA, small for gestational age.
