# Supplementary material for: One test for all: whole exome sequencing significantly improves the diagnostic yield in growth retarded patients referred for molecular testing for Silver–Russell syndrome
Source: Orphanet J Rare Dis. 2021 Jan 22;16:42. doi: 10.1186/s13023-021-01683-x (PMC7821667; doi:10.1186/s13023-021-01683-x)
Supplement: Supplementary file 2 — Additional file 2. Table 2 List of genes used for targeted NGS analysis. It should be noted that the list is based on that from Meyer et al. [7] and does not include the recently identified SRS gene PLAG1 [14]. [file 13023_2021_1683_MOESM2_ESM.docx]

**Supplementary File 2**

List of genes in the targeted multigene panel

| *ABCC8* | *DLK1* | *IGFBP3* | *RTL1* |
| --- | --- | --- | --- |
| *ANKRD11* | *GRB10* | *KCNJ11* | *SGCE* |
| *BLM* | *HMGA2* | *MEST* | *SRCAP* |
| *CCDC8* | *IGF1* | *NBN* | *TRIM37* |
| *CDKN1C* | *IGF1R* | *NSD1* |  |
| *COPG2* | *IGF2* | *OBSL1* |  |
| *CUL7* | *IGF2BP3* | *PIK3R1* |  |
