# Supplementary material for: One test for all: whole exome sequencing significantly improves the diagnostic yield in growth retarded patients referred for molecular testing for Silver–Russell syndrome
Source: Orphanet J Rare Dis. 2021 Jan 22;16:42. doi: 10.1186/s13023-021-01683-x (PMC7821667; doi:10.1186/s13023-021-01683-x)
Supplement: Supplementary file 3 — Additional file 3. Table 3: Overview on the availability of clinical data for the patients from the three different NGS strategies. [file 13023_2021_1683_MOESM3_ESM.docx]

**Suppl. Table 3:** Overview on the availability of clinical data for the patients from the three different NGS strategies.

| Panel | total | 47 |
| --- | --- | --- |
|  | NH-CSS applicable | 34 |
|  | NH-CSS positive | 14 |
|  | NH-CSS not applicable | 13 |
| Index-based WES | total | 31 |
|  | NH-CSS applicable | 23 |
|  | NH-CSS positive | 7 |
|  | NH-CSS not applicable | 8 |
| Trio-based WES | total | 16 |
|  | NH-CSS applicable | 12 |
|  | NH-CSS positive | 5 |
|  | NH-CSS not applicable | 4 |
