# Supplementary material for: One test for all: whole exome sequencing significantly improves the diagnostic yield in growth retarded patients referred for molecular testing for Silver–Russell syndrome
Source: Orphanet J Rare Dis. 2021 Jan 22;16:42. doi: 10.1186/s13023-021-01683-x (PMC7821667; doi:10.1186/s13023-021-01683-x)
Supplement: Supplementary file 4 — Additional file 4. File 4: Patients description. [file 13023_2021_1683_MOESM4_ESM.docx]

**Supplementary File 4: Patient description**

**Patient 1: *PLAG1* - NM_002655.2:c.599dup, p.(Arg201Profs*52)**

The little girl was born SGA at gw 36, postnatal growth retardation persisted and relative macrocephaly became obvious. She showed a triangular face with protruding forehead and micrognathia. No further features were noted at the age of 1 7/12 years. The 1bp-insertion in *PLAG1* occurred de-novo, family history was empty. Clinical scoring revealed four out of six parameters of the NH-CSS.

**Patient 2: *IGF2* - NM_001127598:c.381T>G, p.(Cys127Trp)**

Heterozygosity for the paternally inherited missense variant was detected in female sibs. According to the ACMG criteria (1), the variant should be classified as variant of uncertain significance, but due the imprinting status of the gene and the segregation in the family it can be regarded as likely pathogenic. The patient and her sister were referred for SRS diagnostics as they showed severe intrauterine growth retardation. The elder sister was born SGA at 37 gw, and growth retardation persisted. Nasogastric feeding was required for three months. Craniofacial aspects comprised a small triangular face with frontal bossing, deep set eyes, a depressed nasal bridge, a long philtrum and downturned corners of the mouth. Asymmetry of the lower limbs was documented. A skeletal maturation study showed delayed bone age about 9 months less than chronological age. Echocardiography showed a bicuspid aortic valve with mild stenosis. Her insulin-like growth factor-1 (IGF-1) level was normal at 20.8 nmol/l (ref. 11-58.5). Thyroid function tests were normal, and her celiac screen was negative. Clinical scoring revealed five out of six parameters of the NH-CSS , but relative macrocephaly was not present.

The younger sister showed a similar phenotype. Intrauterine growth retardation and oligohydramnios were documented. Nasogastric feeding for the first five days of life was needed. Cardiac ultrasound revealed a ventricular and atrial septal defect, and a bicuspid aortic valve. She also had numerous characteristic dysmorphic features such as a relatively large head, protruding forehead, triangular face, a smooth philtrum, downturned corners of the mouth, small chin, micrognathia, clinodactyly of the fifth finger on both hands, and asymmetry of the lower limbs. Four out of four available criteria of the NH-CSS were fulfilled.

**Patient 3: *HMGA2* - NM_003483.4:c.111+1G>T, p.?**

Heterozygosity for the de-novo splice-site variant was detected in a four year-old boy. Clinical diagnosis revealed 4 out of 6 parameters of the NH-CSS, the patient has already been reported (Hübner et al., in press).

**Patient 4: *HMGA2 -* NM_003483.4:c.239C>T, p.(Pro80Leu)**

In a consanguineous (1st cousin) Egyptian family homozygosity for the missense variant was identified in two siblings with severe short stature. All four documented NH-CSS parameters were present. The family has recently been described by Hübner et al. (revised version submitted) and the variant was already classified as pathogenic.

**Patient 5: *IGF1R* - NM_000875:c.3530G>A, p.(Arg1177His)**

The patient was born SGA, but further progression of growth retardation was documented. At the age of 1 11/12 years severe microcephaly was reported. The NH-CSS resulted in 3 out of 6 parameters. In addition to retarded growth, café-au-lait spots were present. The patient was heterozygous for a missense variant which has recently been described as pathogenic(2). The variant was inherited from the father who was of normal height (172 cm) and did not show any further features. However, *IGF1R* variants show a broad clinical variability (3) and therefore the variant in the patient can be regarded as pathogenic.

**Patient 6: *ORC1* - NM_004153.3:c1996C>T / NM_004153.3:c.692del, p.(Arg666Trp) / p.(Pro231Glnfs*12)**

Compound heterozygosity for missense and frameshift variant in *ORC1* were identified in a 30-year-old woman suffering from severe growth retardation. Both variants have already been listed in dbSNP and with very low frequencies in gnomAD (c.1996C>T: rs201253919, gnomAD: A:0.000048; c.692del: rs1362231446, gnomAD: delG:0.000004), the missense variant has been classified as pathogenic before (ClinVar: RCV000023160.3). Clinical scoring revealed three out of six criteria of the NH-CSS, but neither protruding forehead nor relative macrocephaly were present.

**Patient 7: *OBSL1* - NM_015311.2:c.1382G>A, p.(Trp461*) / arr[hg19] 2q35(220300895_220596562)x1**

Biometric data were not available at birth, but the family reported SGA with a relative large head. Relative macrocephaly then became obvious at the age of 3 8/12 years, whereas growth retardation persisted. In the course of the molecular diagnostic workup, the 29 kb deletion affecting *OBSL1* was detected first, and WES then identified the nonsense variant. NH-CSS was positive (four out of six parameters).

**Patient 8: *MBTPS1* - NM_003791.3:c.1094A>G, p.(Asp365Gly)**

Homozygosity for the missense variant was identified in a patient, born to distantly related parents. He was born SGA with relative macrocephaly, and postnatally growth retardation persisted. In childhood, the facial gestalt corresponded to that of SRS, though a protruding forehead was not present (NH-CSS: four out of six parameters). Further clinical and biochemical data have been recently submitted (Meyer et al., unpublished data). The variant c.1094A>G has already been reported as pathogenic (dbSNP: rs1226321681; gnomAD: ALL: 0.00040%, ClinVAR: RCV000767393.1(4).

**Patient 9: *FANCA* - NM_000135.2:c.2851C>T / c.2222+1G>T; p.(Arg951Trp) / p.?**

The girl was the first child of healthy parents, she was born SGA at 35 gw. She showed esophageal atresia type II. Growth retardation persisted at the age of 3 3/12 years and SRS was diagnosed due to a triangular face with a protruding forehead and micrognathia, a long philtrum, and downturned corners of the mouth. Hands and feet were small, clinodactyly V and brachydactyly V were documented. In total, four out of six NH-CSS criteria were fulfilled, but did not include relative macrocephaly. At the age of 11 ¾ years, Fanconi anemia was diagnosed clinically.

Molecular testing showed compound heterozygosity for two already known pathogenic variants in the *FANCA* gene, a missense variant (ClinVar: RCV000466964.2, RCV00069024.1) and a splice site variant. The splice site variant could also be detected in the mother of the patient whereas a paternal DNA sample was not available for testing.

**Patient 10: *NF1* - NM_001042492.2:c.5488C>T; p.(Arg1830Cys)**

Due to preeclampsia, delivery was induced and the boy was born after Caesarean section at gw 25.5 (birth weight 620 g (-1.33SDS), length 32 cm (-0.7 SDS), OFC 22.5 cm (-1.11 SDS)). Umbilical hernia, persistent ductus arteriosus (PDA) and hypospadias were reported. Feeding difficulties persisted and gastric tube feeding was required at the age of 6 2/12 years. Severe growth retardation was obvious (weight 12.65 kg (-5.08 SDS), length (105 cm (-2.86 SDS), OFC 47.5 cm (-3.65 SDS). Bone age at 6 years was slightly retarded (corresponding to an age of 4 years). A triangular face with a prominent forehead lead to the suspicion diagnosis of SRS. One café-au-lait spot was detected, the patient showed a mild developmental delay. NH-CSS was three out of five items. Family history was empty, the mother did not show café-au-lait spots

In the patient and his mother heterozygosity for a missense variant in the *NF1* gene was detected which has already been identified as pathogenic. It is a loss-of-function variant which is associated with a variable neurofibromatosis type 1 pigmentary phenotype(5).

**Patient 11: *FGD1 -*** **NM_00046.3:c.2761C>T, p.(Arg921*)**

The boy was born at term with a birth weight of 2700 g (-2.13 SDS), length of 48 cm (-1.99 SDS) and an OFC of 33 cm (-2.03 SDS). At the age of 13 3/12 years height was 148.8 cm (-1.49 SDS), weight 38 cm (-1.26 SDS) and OFC 52 (-2.11 SDS). The patient showed a protruding forehead with a triangular face, and a clinodactyly V. Due to the craniofacial gestalt, the diagnosis of SRS was discussed. Development was reported as normal. NH-CSS was negative (2 out of 5 parameters).

WES analysis revealed hemizygosity for a known de-novo nonsense substitution in the *FGD1* gene (ClinVar: RCV000210282.1), ACMG classification classified the variant as likely pathogenic. Variants in *FGD1* cause the X chromosomal Aarskog-Skott syndrome(6). Due to the de-novo occurrence a pathogenic impact can be delineated.

**Patient 12: *CNOT3* - NM_014516.3:c.658G>T, p.(Glu220*)**

The index patient was referred for molecular SRS testing because of SGA and persisting growth retardation, but data on postnatal growth were not available. Development was delayed. The nonsense variant had occurred de-novo and was then inherited to two affected daughters (for further details see Meyer et al., submitted).

**Patient 13: *KMT2C* - NM_170606.2:c.11023C>T, p.(Gln3675*)**

The patient was born at term, weight, length and OFC were low but within the normal range. After birth, growth retardation was observed. His facial gestalt was described as triangular, but a protruding forehead was not observed. Psychomotoric development was slightly delayed, but the patient finished secondary education. An attention deficit disorder was diagnosed. NH-CSS was negative (1 out of 6 items). The nonsense variant was not listed in public databases (dbSNP, ClinVar, gnomAD). Pathogenicity is likely as the reading frame is interrupted by a premature stop codon. Pathogenic variants in *KMT2C* are associated with Kleefstra syndrome 2(7), and the phenotype of our patient was in accordance with the molecular finding.

**Patient 14: *PTEN* - NM_000314.7:c.518G>A, p.(Arg173His)**

Patient 14 was ascertained because of postnatal growth retardation (a the age of 6 6/12 years: 21.8 kg (-0.34 z), 110 cm (-2.25 z)) and triangular face. Developmental delay was noted. After starting with growth hormone treatment, growth normalised (see table 1). The patient was heterozygous for a missense variant in the *PTEN* gene, this variant has already been reported in a cohort of individuals with autism and developmental delay(8). The patient met only one out of five NH-CSS criteria.

**Case 15: *PTPN11* - NM_002834.4:c.1508G>A, p.(Gly503Glu)**

The patient was referred for SRS testing because of SGA, persistent growth retardation and a triangular face. His behaviour was documented as aggressive, and developmental delay was reported. Due to the lack of further clinical data, the NH-CSS could not be applied. The missense variant is already listed in ClinVar and has been classified as pathogenic/likely pathogenic (RCV000532971.1, RCV000033548.6).

**References**

1. Richards S, Aziz N, Bale S, Bick D, Das S, Gastier-Foster J, et al. Standards and guidelines for the interpretation of sequence variants: a joint consensus recommendation of the American College of Medical Genetics and Genomics and the Association for Molecular Pathology. Genet Med. 2015;17(5):405-24.

2. Gkourogianni A, Andrade AC, Jonsson BA, Segerlund E, Werner-Sperker A, Horemuzova E, et al. Pre- and postnatal growth failure with microcephaly due to two novel heterozygous IGF1R mutations and response to growth hormone treatment. Acta Paediatr. 2020.

3. Walenkamp MJE, Robers JML, Wit JM, Zandwijken GRJ, van Duyvenvoorde HA, Oostdijk W, et al. Phenotypic Features and Response to GH Treatment of Patients With a Molecular Defect of the IGF-1 Receptor. J Clin Endocrinol Metab. 2019;104(8):3157-71.

4. Kondo Y, Fu J, Wang H, Hoover C, McDaniel JM, Steet R, et al. Site-1 protease deficiency causes human skeletal dysplasia due to defective inter-organelle protein trafficking. JCI Insight. 2018;3(14).

5. Rojnueangnit K, Xie J, Gomes A, Sharp A, Callens T, Chen Y, et al. High Incidence of Noonan Syndrome Features Including Short Stature and Pulmonic Stenosis in Patients carrying NF1 Missense Mutations Affecting p.Arg1809: Genotype-Phenotype Correlation. Hum Mutat. 2015;36(11):1052-63.

6. Griffin LB, Farley FA, Antonellis A, Keegan CE. A novel FGD1 mutation in a family with Aarskog-Scott syndrome and predominant features of congenital joint contractures. Cold Spring Harb Mol Case Stud. 2016;2(4):a000943.

7. Koemans TS, Kleefstra T, Chubak MC, Stone MH, Reijnders MRF, de Munnik S, et al. Functional convergence of histone methyltransferases EHMT1 and KMT2C involved in intellectual disability and autism spectrum disorder. PLoS Genet. 2017;13(10):e1006864.

8. Varga EA, Pastore M, Prior T, Herman GE, McBride KL. The prevalence of PTEN mutations in a clinical pediatric cohort with autism spectrum disorders, developmental delay, and macrocephaly. Genet Med. 2009;11(2):111-7.
